# A Fast Map Merging Algorithm in the Field of Multirobot SLAM

**DOI:** 10.1155/2013/169635

**Published:** 2013-11-02

**Authors:** Yanli Liu, Xiaoping Fan, Heng Zhang

**Affiliations:** ^1^School of Information Science and Engineering, Central South University, Changsha 410075, China; ^2^School of Information Engineering, East China Jiaotong University, Nanchang 330013, China

## Abstract

In recent years, the research on single-robot simultaneous localization and mapping (SLAM) has made a great success. However, multirobot SLAM faces many challenging problems, including unknown robot poses, unshared map, and unstable communication. In this paper, a map merging algorithm based on virtual robot motion is proposed for multi-robot SLAM. The thinning algorithm is used to construct the skeleton of the grid map's empty area, and a mobile robot is simulated in one map. The simulated data is used as information sources in the other map to do partial map Monte Carlo localization; if localization succeeds, the relative pose hypotheses between the two maps can be computed easily. We verify these hypotheses using the rendezvous technique and use them as initial values to optimize the estimation by a heuristic random search algorithm.

## 1. Introduction

Recent advances in mobile robotics have allowed autonomous systems to be involved in many successful applications including planetary exploration, search and rescue, surveillance, and other service scenarios [1].

For the purpose of successfully accomplishing a generic task, a main prerequisite for a mobile robot, deployed in an unknown area, is the ability to autonomously navigate, exploiting the information acquired through the joint estimation of its positions and a model of the surrounding environment. The problem of estimating both the robot pose and the environment representation is usually defined as simultaneous localization and mapping (SLAM) [2, 3]. While the maturity of SLAM in single robot scenarios is recognized in many recent works, a challenging issue is to extend these approaches to multirobot scenarios [4–7].

Recently, in order to cope with dynamic environment and multiple tasks, a lot of researches have been presented in multiagent and multiple distributed autonomous robotic systems. Each robot has its own task, such as building a map of local position. Moreover, they have to combine their data into shared maps. Using shared maps, robots coordinate their exploration strategies to maximize the efficiency of exploration. Multiple autonomous mobile robots can complete the task through cooperation and give a more accurate map by data fusion. The robots coordinate their exploration strategies to maximize the efficiency of their exploration using these shared maps.

Fenwick et al. extended single robot SLAM algorithm based on EKF to the multirobot [8–10]. Howard extended single-robot SLAM algorithm based on particle filter to multi-robot applications [11]. Thrun put forward multi-robot hybrid map building method that combines fast maximum likelihood map growing with a Monte Carlo localizer [12]. Thrun [10] points out that the key to multi-robot map building is to determine the initial position and orientation of each single robot. However, most current algorithms bypass the problem, either some algorithms assume that the road signs in the environment are identified, or some algorithms assume that each robot starting from a similar position, so that their initial map has large coverage area. Ko et al. [13] specialize in the methods by which the robots determine relative pose in environmental exploration. Each robot builds its own maps, and meanwhile, estimates the pose of other robots in its own map. If the estimation probability reaches the higher accuracy, two robots rendezvous [14] to confirm the pose between them, and this paper also adopts a similar approach. Another strategy is to assume that the robots can be cross-observed to determine the relative pose between them [13, 15]. The deficiency of the method is that each robot in the process of movement must be “accidentally” encounter.

In this paper we present a novel fast and accurate algorithm for merging multiple maps represented as occupancy grids. The main idea is as follows: make a virtual mobile robot in one partial map and control its move and measure in the map. At the same time, these simulated data are used as information sources in the other map to do partial map Monte Carlo localization [16]; if localization succeeds, the relative pose hypotheses between the two maps can be computed easily. In order to speed up the process, we can constrain the virtual robot move along the local topological edge of the partial map, so in the process of Monte Carlo localization within the partial map, the initial random samples only are generated near the edges of the corresponding topology of the other partial map. Prior knowledge of the relative pose between maps or robots is not demanded in the method, which enhances autonomous exploration and multi-robot SLAM.

## 2. Concept of Map Merging

The research on map merging is limited, and the previous work focused on feature-based approaches [17–19], which rely on special landmarks that can be recognized through properly processing the data gathered by the robots. For example, the topological maps are graphs in which vertices represent recognizable places such as doorways, corridors, and different types of corner and edges represent the passage connecting two places. Merging map A and map B turns into searching subgraphs with the same structure in map A and map B. Our goal is to merge maps which are not based on features, but rather on occupancy grids. For sake of clearness, we formally define the several concepts [20].

**Definition 1**

Let *N* and *M* be two positive real numbers. A *N* × *M* map is a function. Consider

(1) $$\begin{array}{c} m:\left[1,N\right]\times \left[1,M\right]\to R, \end{array}$$

where *I*_*N*×*M*_ denoting the set of *N* × *M* maps.

Discrimination is needed when a map is processed in the practical application, and the map is represented as a matrix with *N* rows and *M* columns. The function *m* is a confidence model in the map. For example, one could assume that if the value of *m*(*x*, *y*) is positive, the point (*x*, *y*) in the map is free, while a negative value represents the point (*x*, *y*) as occupied. The absolute value represents the degree of belief. Especially, we assume that if the value of *m*(*x*, *y*) is zero, the point (*x*, *y*) is unknown. The definition is consistent with the grid map. In the following, the planar transformation is defined, which will be used to find the best matching between two partial maps.

**Definition 2**

*t*_*x*_, *t*_*y*_, and *t*_*θ*_ are three real numbers; the pose transformation function consists of *t*_*x*_, *t*_*y*_, and *t*_*θ*_ is defined as follows:

(2) $$\begin{array}{c} T_{\left(t_{x},\;t_{y},\;t_{\theta }\right)}\left(x,y\right):R^{2}\to R^{2}, \end{array}$$

where *t*_*x*_, *t*_*y*_, and *t*_*θ*_ are three real numbers. It is defined as follows:

(3) $$\begin{array}{c} T_{\left(t_{x},\;t_{y},\;\theta \right)}\left(x,y\right)=\left[\begin{array}{ccc} 1 & 0 & 0\\ 0 & 1 & 0 \end{array}\right]\left[\begin{array}{ccc} \cos\theta & -\sin\theta & t_{x}\\ \sin\theta & \cos\theta & t_{y}\\ 0 & 0 & 1 \end{array}\right]\left[\begin{array}{c} x\\ y\\ 1 \end{array}\right]. \end{array}$$

**Definition 3**

*m*_1_ and *m*_2_ are the two maps of *I*_*N*×*M*_; the coverage between *m*_1_ and *m*_1_ is defined as follows:

(4) $$\begin{array}{c} \omega \left(m_{1},m_{2}\right)=\sum_{i=1}^{N}\sum_{j=1}^{M}e\left(m_{1}\left[i,j\right],m_{2}\left[i,j\right]\right), \end{array}$$

where

(5) $$\begin{array}{c} e\left(a,b\right)=\{\begin{array}{cc} 1, & \text{if}\;a=b,\\ 0, & \text{otherwise}. \end{array} \end{array}$$

The coverage function is used to measure the degree of the match between two maps. Ideally, the map, which the two robots build, is entirely consistent with the physical environment and covers the entire environment space. In this case, the coverage function is *ω*(*m*_1_, *m*_2_) = *N* × *M*; it is maximum.

According to the above definitions, we can formally make definitions on map merging.

**Definition 4**

Given two maps *m*_1_ and *m*_2_, pose transformation function *T*_(*t*_*x*_,*t*_*y*_,*t*_*θ*_)_(*x*, *y*) is searched to maximize the function *ω*(*m*_1_, *T*_(*t*_*x*_,*t*_*y*_,*t*_*θ*_)_ *m*_2_).

From the previous definition, the key to map merging is to search the optimal pose transformation *T*_(*t*_*x*_,*t*_*y*_,*t*_*θ*_)_. The preliminary estimations of the transformation are got by the Monte Carlo localization in the partial map [16]. In order to improve computational efficiency, we adopt the sampling method with confined area. The heuristic random search algorithm is used to further optimize the pose transformation.

## 3. Fast Monte Carlo Global Localization

### 3.1. Topological Information of a Map

The topological information of a map is the abstraction of environmental information. Vertices represent discrete places in the environment, and edges represent paths connecting these places. The information can be obtained by generalized Voronoi graph (GVG) algorithm [21] or thinning algorithm [22, 23]. Useless boundaries and virtual intersections for navigation are generated in GVG algorithm, the thinning method does not generate this information because it is based on probabilistic framework. It is robust for the sensor noise and various environments.

**Figure 1:**
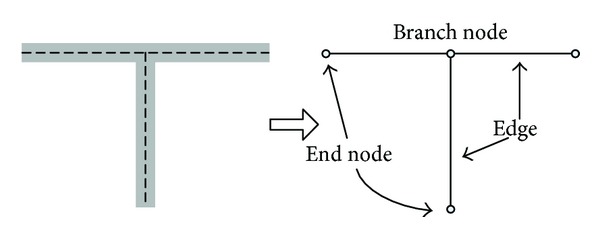
Concept of thinning.

Thinning algorithm is the method that extracts the image skeleton in the digital image processing algorithm.  Figure 1 illustrates the concept of thinning. The object on the left figure can be described well by the structure composed of connected lines (i.e., “T” shape design with thin lines on the right figure). Note that connectivity of the structure is still preserved even with thin lines. For mobile robots, the connecting lines can be used as collisionless paths of the robot navigation.

**Figure 2:**
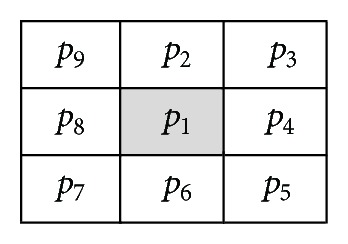
Centre grid *p*_1_ and its 8 neighbor grids.

In the paper, eight neighbour grid operators are used to solve the skeleton of free areas in grid map. Centre grid *p*_1_ and 8 neighbor grids (*p*_2_ ~ *p*_9_) are described in Figure 2. “0” indicates that the grid is empty and “1” indicates the grid is occupied. For the occupied grid *p*_1_, if its eight neighbour grids satisfy the thinning condition, grid *p*_1_ is change into empty because it is not a part of the skeleton.

*Thinning Condition 1*. Consider

1. 2 ≤ *N*(*p*_1_) ≤ 6,
2. *S*(*p*_1_) = 1,
3. *p*_2_ · *p*_4_ · *p*_6_ = 0,
4. *p*_4_ · *p*_6_ · *p*_8_ = 0.

*Thinning Condition 2 *

1.   and (2) are the same as Thinning Condition 1,
2.  
*p*_2_ · *p*_4_ · *p*_8_ = 0,
3.  
*p*_2_ · *p*_6_ · *p*_8_ = 0.

Here,

(6) $$\begin{array}{c} p_{i}=\{\begin{array}{cc} 0, & \text{grid}\;\;p_{i}\;\;\text{is}\;\;\text{empty},\\ 1, & \text{grid}\;\;p_{i}\;\;\text{is}\;\;\text{occupied}. \end{array} \end{array}$$

*N*(*p*_1_) denotes the number of occupied grids of 8 neighbours, and it is defined as follows:

(7) $$\begin{array}{c} N\left(p_{1}\right)=\sum_{i=2}^{9}p_{i}. \end{array}$$

*S*(*p*_*i*_) denotes times of the change from 0 to 1 in the sequence *p*_2_, *p*_3_,…, *p*_8_, *p*_9_.

### 3.2. The Sample Space Limitation

The map skeleton is constructed for designating confined sample area and navigation path. If robot moves along bisector of the obstacles (approximately along the skeleton), it has more opportunities to collect more environment information.

The performance of MCL is heavily dependent on density of particles if the other conditions are the same. The sample space is usually very large in the stages of MCL global localization and processing the kidnapped robot problem (the worst case is all free areas in the environmental map). A lot of particles are required in order to localize successfully, and the request of real time is not met. However, if we could acquire the relevant knowledge about pose of robot in advance, for example, we are sure that robot is located in the area that is much smaller than free area in the map, we can only sample in this area, which improves the speed of the algorithm.

## 4. Relative Pose Estimation of Maps

### 4.1. Hypothesis Generation

**Figure 3:**
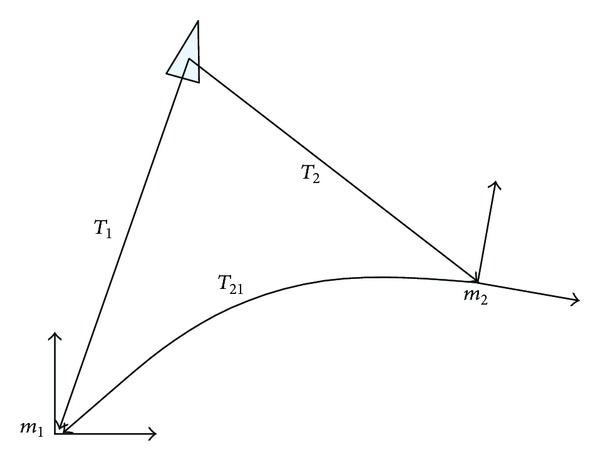
Map merging coordinate diagram.

The simplest situation is considered: two complete maps *m*_1_ and *m*_2_ (their coordinates are different in global coordinates) in the same closed environment *Ω* save two parts of information: grid occupancy information and skeleton of corresponding free area. If a robot constantly moves along the skeleton of free area in environment *Ω* and observes the environment at the same time, MCL global localization is used to localize the robot in map *m*_1_ and map *m*_2_. Suppose the location estimations of robot in two maps are *ξ*_*t*_^(1)^ and *ξ*_*t*_^(2)^, respectively, at time *t*, as shown in Figure 3, the following formula can be concluded:

(8) $$\begin{array}{c} T_{1}=T_{21}\cdot T_{2}, \end{array}$$

where *T*_1_ and *T*_2_ are corresponding homogeneous transformation matrixes to *ξ*_*t*_^(1)^ and *ξ*_*t*_^(2)^, respectively. *T*_21_ is homogeneous transformation matrix that the coordinates of map *m*_2_ is relative to one of map *m*_1_.

According to (4), the relative pose between *m*_1_ and *m*_2_ is as follows:

(9) $$\begin{array}{c} T_{21}=T_{1}\cdot T_{2}^{-1}. \end{array}$$

**Figure 4:**
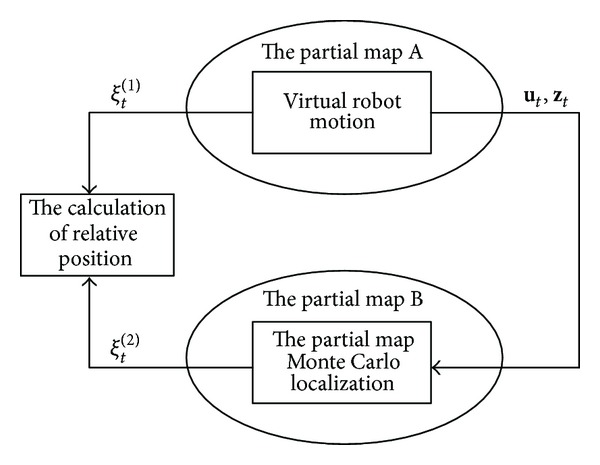
Calculation of relative pose of maps based on virtual robot motion.

The specific approach is put forward in the paper. A robot is simulated to move along the skeleton of the partial map *m*_1_ and constantly observe the map, which is obtained by a robot using the single-robot-SLAM algorithm. At the same time, these simulated data are used as information sources to do Monte Carlo localization for partial map in the map *m*_2_. The localization process is carried out until the particles converge to one or more clusters. Because the simulation data is obtained near the map skeleton, the initial particles only need to be generated near the skeleton of *m*_2_ in the process of MCL in the map *m*_2_. In this way the initial pose estimation between *m*_1_ and *m*_2_ is got quickly.  Figure 4 shows the model of the method.

### 4.2. Hypothesis Verification

**Figure 5:**
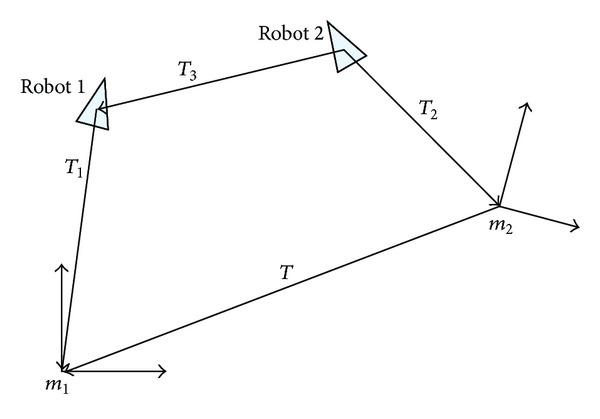
Relative pose of robots.

From the previous method, we get the preliminary estimation to the relative pose between two partial maps *m*_1_ and *m*_2_ which were constructed by Robot1 and Robot2, respectively. The estimation may have one or more peak values, which are supposed to be as relative pose hypotheses between *m*_1_ and *m*_2_. Suppose that homogeneous transformation matrix *T* is one of these hypotheses, as show in Figure 5, the homogeneous transformation matrix *T*_3_ of the two robots' relative pose can be calculated with *T*, *T*_1_, and *T*_2_ as follows:

(10) $$\begin{array}{c} T_{3}=T_{1}^{-1}\cdot T\cdot T_{2}. \end{array}$$

We can use *T*_3_ to path planning two robots' paths in the map *m*_1_ and make them meet as far as possible. The plan is operated on Robot1, and the results are sent to Robot2 in order to collaborate with each other. If the two robots can meet according to the path planning, the relative pose hypothesis *T* is true, and we go to the stage of estimation optimization; otherwise we test the next hypothesis. If all hypotheses are false, the robot still constructs map independently (single-robot SLAM), waiting for the next operation of map merging.

## 5. Estimation Optimization

After the previous steps, the preliminary estimation to the relative pose of map is got. Based on the research of literature [24], the relative pose is further optimized. The concrete optimization process is as follows.

### 5.1. Dissimilarity Measurement Function of Map

From the definition of map merging, the map merging problem can be seen as an optimization problem, whose optimization function is *ω*(). It is the problem that *ω*() is directly used as optimization function. Because the values of *ω*(*m*_1_, *T*_(*t*_*x*_,*t*_*y*_,*t*_*θ*_)_(*m*_2_)) are arbitrarily leap with continuous changes of variables (*t*_*x*_, *t*_*y*_, *t*_*θ*_), the function *ω*() delivers no effective gradients to do optimization like hill-climbing algorithm. The dissimilarity function is as follows:

(11) $$\begin{array}{c} \psi \left(m_{1},m_{2}\right)=\sum_{c\in C}\left[d\left(m_{1},m_{2},c\right)+d\left(m_{1},m_{2},c\right)\right] \end{array}$$

with

(12) $$\begin{array}{c} d\left(m_{1},m_{2},c\right)=\frac{\sum_{m_{1}[p_{1}]=c}\min\left\{md\left(p_{1},p_{2}\right)\;|\;m_{2}\left[p_{2}\right]=c\right\}}{{\#}_{c}\left(m_{1}\right)}, \end{array}$$

where *C* denotes grid range of map *m*_1_ and map *m*_2_, *m*_1_[*p*_1_] denotes the value of grid *p*_1_ in map *m*_1_, *md*(*p*_1_, *p*_2_) = |*x*_1_ − *x*_2_| + |*y*_1_ − *y*_2_| denotes the Manhattan-distance between points *p*_1_ and *p*_2_, and #_*c*_(*m*_1_) = #{*p*_1_ | *m*_1_[*p*_1_] = *c*} denotes the number of grids with value *c* in map *m*_1_.

To simplify the calculation, the grids' value of the map is marked as “free” or “occupied” or “unknown” according to the predefined threshold. Only occupied and free grids are considered for computing dissimilarity function, so *C* = {occ, free}. In order to compute the dissimilarity function in linear time, a so called distance-map is introduced. Distance-map *dmap*_*c*_[*x*_1_][*y*_1_] denotes the Manhattan distance between the grid *p*_1_ = (*x*_1_, *y*_1_) in map *m*_1_ and a grid which is the nearest point to *p*_1_ with value *c* in map *m*_2_:

(13) $$\begin{array}{c} dmap_{c}\left[x_{1}\right]\left[y_{1}\right]=\min\left\{md\left(p_{1},p_{2}\right)|m_{2}\left[p_{2}\right]=c\right\}. \end{array}$$

The concrete calculation process for *dmap*_c_ is shown in Algorithm 1.

Using the distance-map *dmap*_*c*_, we can calculate *d*(*m*_1_, *m*_2_, *c*) with Algorithm 2.

### 5.2. Random Walk Optimization

The basic idea of random walk optimization is to search in the given solution space using the way of random walk. At each step a random solution is generated and the corresponding heuristic rule of next step is computed. The concrete process is shown in Algorithm 3. And a more detailed description is in the literature [24].

## 6. Experimental Results

**Figure 6:**
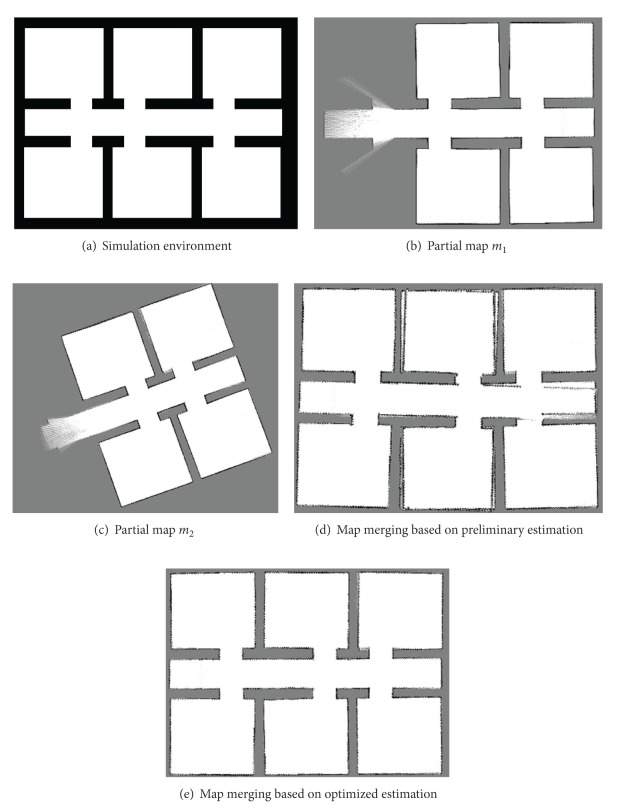
Experimental results.

We perform experiment based on the map merging algorithm proposed in this paper. The process of experiment is as follows. Firstly, grid map of the identical simulation environment (Figure 6(a)) is constructed by using SLAM algorithm twice, as shown in Figures 6(b) and 6(c). Then, the relative pose between two partial maps is calculated using the method proposed in the paper. Finally, the results of map merging are illustrated in Figures 6(d) and 6(e).  Figure 6(d) is the result based on calculation of relative pose of maps using virtual robot motion, and its optimized result using random walk optimization is showed in Figure 6(e).

## 7. Conclusions

In the paper, map merging method based on virtual robot motion is proposed in the field of multirobot SLAM. For multi-robot SLAM, there are four kinds of interaction effect between two robots. The first kind is no interaction between two robots. The second kind is hypothesis generation because communication is permitted between robots, but relative pose of other robot is unknown. The third kind is hypothesis verification because communication is permitted between robots, and relative localization hypothesis is generated in the process of hypothesis generation. The forth kind is coordinated exploration because robots have relative pose and can share map and explore environment.

In this paper, a mobile robot is simulated in one map; it moves along the map's skeleton and measures the virtual environment. At the same time, these simulated data are used as information sources in the other map to do partial map Monte Carlo localization; if localization succeeds, the relative pose hypotheses between the two maps can be computed easily. Then, they actively verify one hypothesis using a rendezvous technique. If successful, using the hypothesis as initial value, the estimation is optimized by a heuristic random search algorithm. The algorithm is not only for grid maps but also other types of map. The experimental results have verified the algorithm.

In the future, the corresponding problems, such as network transmission and collaboration of robots, are required to be considered. Cloud robotics is considered to be the next great-leap-forward development of robotics. The method will be improved to apply to cloud robotics.

## Figures and Tables

**Algorithm 1:**
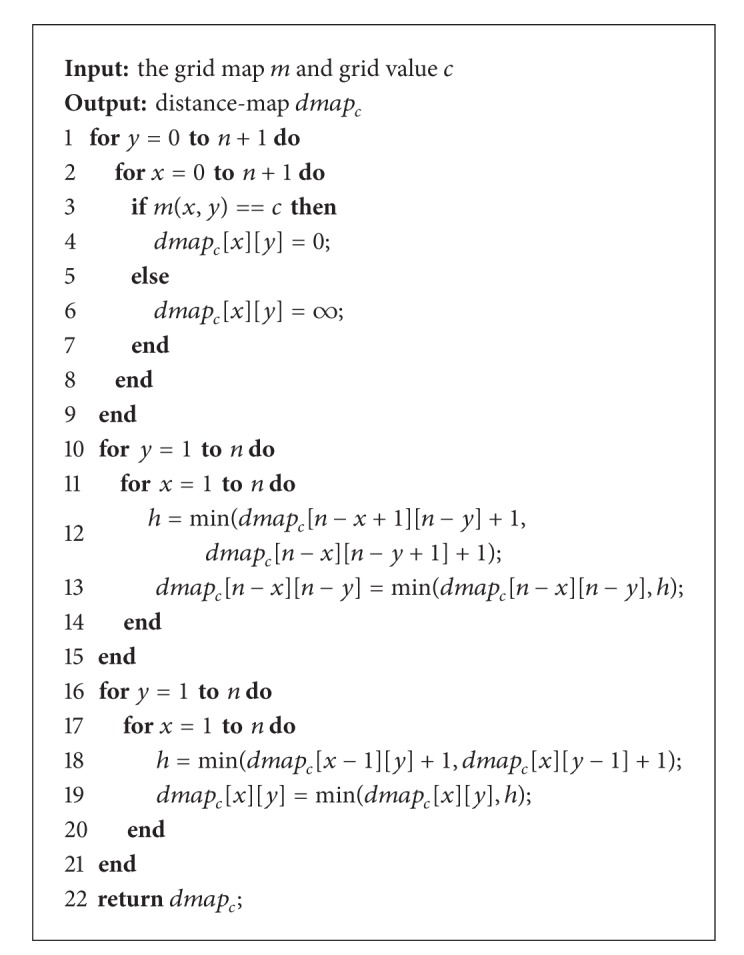
Calculation process of distance-map *dmap*_*c*_ in grid map.

**Algorithm 2:**
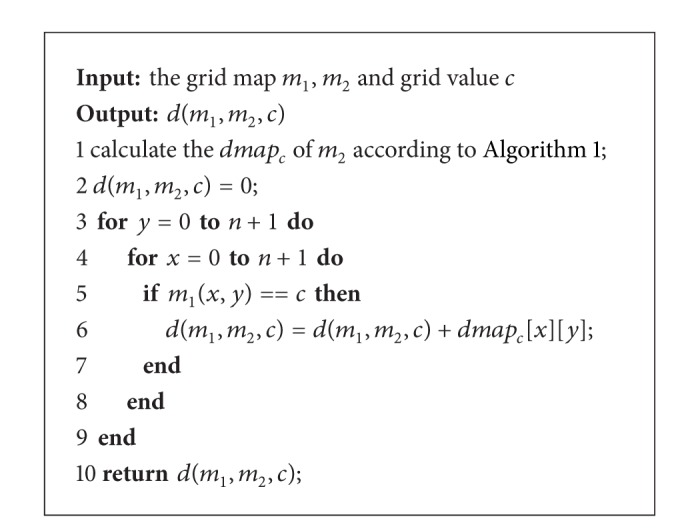
Calculation process of *d*(*m*_1_, *m*_2_, *c*).

**Algorithm 3:**
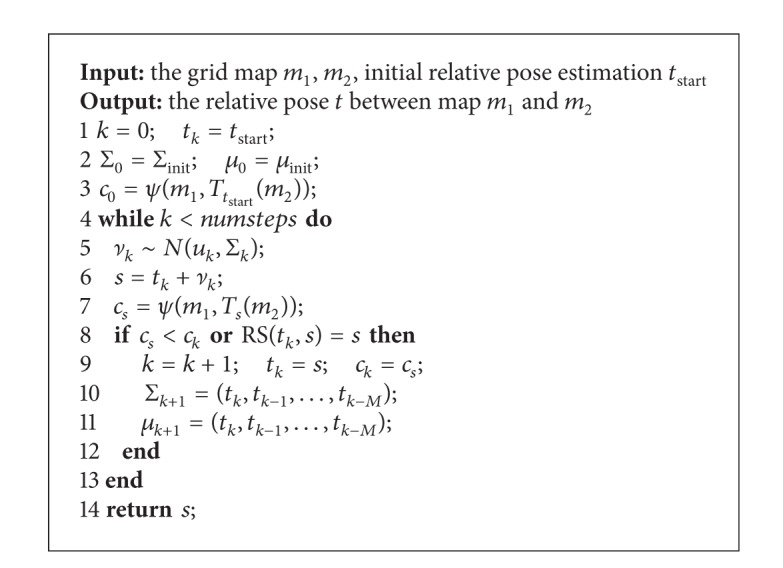
Random walk optimization.
